# Bowel-Associated Dermatosis-Arthritis Syndrome: A Report of a Rare Case

**DOI:** 10.7759/cureus.71217

**Published:** 2024-10-10

**Authors:** Duarte Flor, André Gonçalves, Francisca Morgado, Sandra Barbeiro, Jose C Cardoso

**Affiliations:** 1 Dermatology, Coimbra Local Health Unit, Coimbra, PRT; 2 Gastroenterology, Leiria Local Health Unit, Leiria, PRT

**Keywords:** arthritis, dermatopathology, inflammatory bowel disease, neutrophilic dermatosis, ulcerative colitis (uc)

## Abstract

A 31-year-old female, recently discharged after improvement of an exacerbation of ulcerative colitis, returned to the hospital due to fever, polyarthralgia affecting the knees and ankles, and symmetric violaceous papulopustular lesions on her arms, trunk, and lower limbs. Histological examination revealed superficial dermal edema and perivascular invasion of neutrophils with leukocytoclasia, consistent with neutrophilic dermatosis, and a diagnosis of bowel-associated dermatosis-arthritis syndrome (BADAS) was established.

BADAS is a rare, characteristic neutrophilic dermatosis and arthritis mainly associated with intestinal inflammatory disease due to bacterial overgrowthand subsequent immune complex formation and deposition. Treatment involves a combination of antibiotic therapy and immunosuppression, and prognosis is linked to improvement of the underlying condition.

## Introduction

Bowel-associated dermatosis-arthritis syndrome (BADAS) is a rare condition characterized by neutrophilic dermatosis and arthritis associated with inflammatory bowel disease (IBD), presenting with symptoms such as fever, myalgias, and malaise, alongside skin eruptions on the extremities and trunk. These skin lesions typically start as erythematous macules and progress into purpuric papules, papulopustules, or tender subcutaneous nodules within a short timeframe [1,2].

Originally associated with bowel bypass surgery, BADAS has also been linked to diverticulitis, IBD, and appendicitis [1-8]. The underlying pathophysiology is believed to involve the formation and deposition of immune complexes triggered by antigens from intestinal bacterial overgrowth [3].

One of the challenges in diagnosing BADAS lies in its similarity to other neutrophilic dermatoses, such as Sweet syndrome, pyoderma gangrenosum, and neutrophilic dermatosis of the dorsal hands. Additionally, due to its rare occurrence, many healthcare professionals may not be familiar with the condition, leading to potential misdiagnosis or delayed recognition [3].

We present an unusual case involving BADAS, which was associated with a flare-up and diagnosis of initial ulcerative colitis (UC).

## Case presentation

A previously healthy 31-year-old female patient was admitted to the emergency department due to a persistent 15-day history of bloody diarrhea, abdominal cramps, and elevated fever. She denied any other symptoms, medications, or other relevant prior history. On admission, her laboratory workup showed hypochromic and microcytic anemia (hemoglobin of 10.4 g/dL) and an elevated C-reactive protein (CRP) level (129 mg/dL) with no other remarkable findings (Table 1).

**Table 1:** Complete patient blood and stool testing on admission RDW: red cell distribution width; HLA: human leukocyte antigen

	Value	Unit	Reference min.	Reference max.
Hematology				
Leukocytes	12.6	x10^9/L	4	10
Neutrophils	0.8	x10^9/L	1.8	8
Lymphocytes	0.8	x10^9/L	1.5	6.5
Monocytes	1	x10^9/L	0	0.8
Eosinophils	0.3	x10^9/L	0	0.6
Basophils	0.1	x10^9/L	0	0.2
Erythrocytes	3.8	x10^12/L	3.8	5.8
Hemoglobin	10.1	g/dL	11.5	16
Hematocrit	30.3	%	35	47
Mean corpuscular volume	79.6	fL	80	100
Mean corpuscular hemoglobin	26.6	pg	27	
Mean corpuscular hemoglobin concentration	33.15	g/dL	32	36
RDW	14	%	11	14.5
Platelets	367	x10^9/L	150	400
Sedimentation rate	66	mm		
Biochemistry				
Glucose	4.1	mmol/L	4.1	5.9
Blood urea nitrogen (BUN)	4	mmol/L	2.8	7.2
Creatinine	57	umol/L	45	84
Sodium	139	mmol/L	136	146
Potassium	3.9	mmol/L	3.5	5.1
Clorum	104	mmol/L	101	109
Magnesium	0.88	mmol/L	0.77	1.03
Calcium	2.05	mmol/L	2.2	2.65
Phosphate	0.91	mmol/L	0.81	1.45
Osmolarity	293	mOsm/Kg	275	295
Albumine	34	g/L	35	52
Bilirubin, total	8.7	mmol/L	5	21
Bilirubin, direct	1.7	mmol/L		3.4
Alanine aminotransferase (ALT)	15	U/L	3	34
Aspartate aminotransferase (AST)	14	U/L	15	35
Lactate dehydrogenase (LDH)	134	U/L	100	247
Alkaline phosphatase (ALP)	71	U/L	30	120
C-reactive protein (CRP)	141.3	mg/L		5
Immunology				
Antinuclear antibodies (ANA)	Negative			160
C3	138	mg/dL	90	180
C4	26	mg/dL	10	40
Borrelia burgdorferi, IgG	Negative			
Borrelia burgdorferi, IgM	Negative			
Genetics				
HLA-A	*02*02			
HLA-B	*35*51			
HLA-C	*01*16			
Fecal examination				
Bacteriology				
Gram direct examination	Negative			
Culture	Negative			
Clostridium difficile toxin B	Negative			
Clostridium difficile binary toxin	Negative			
Clostridium difficile 027 strain	Negative			

**Figure 1:**
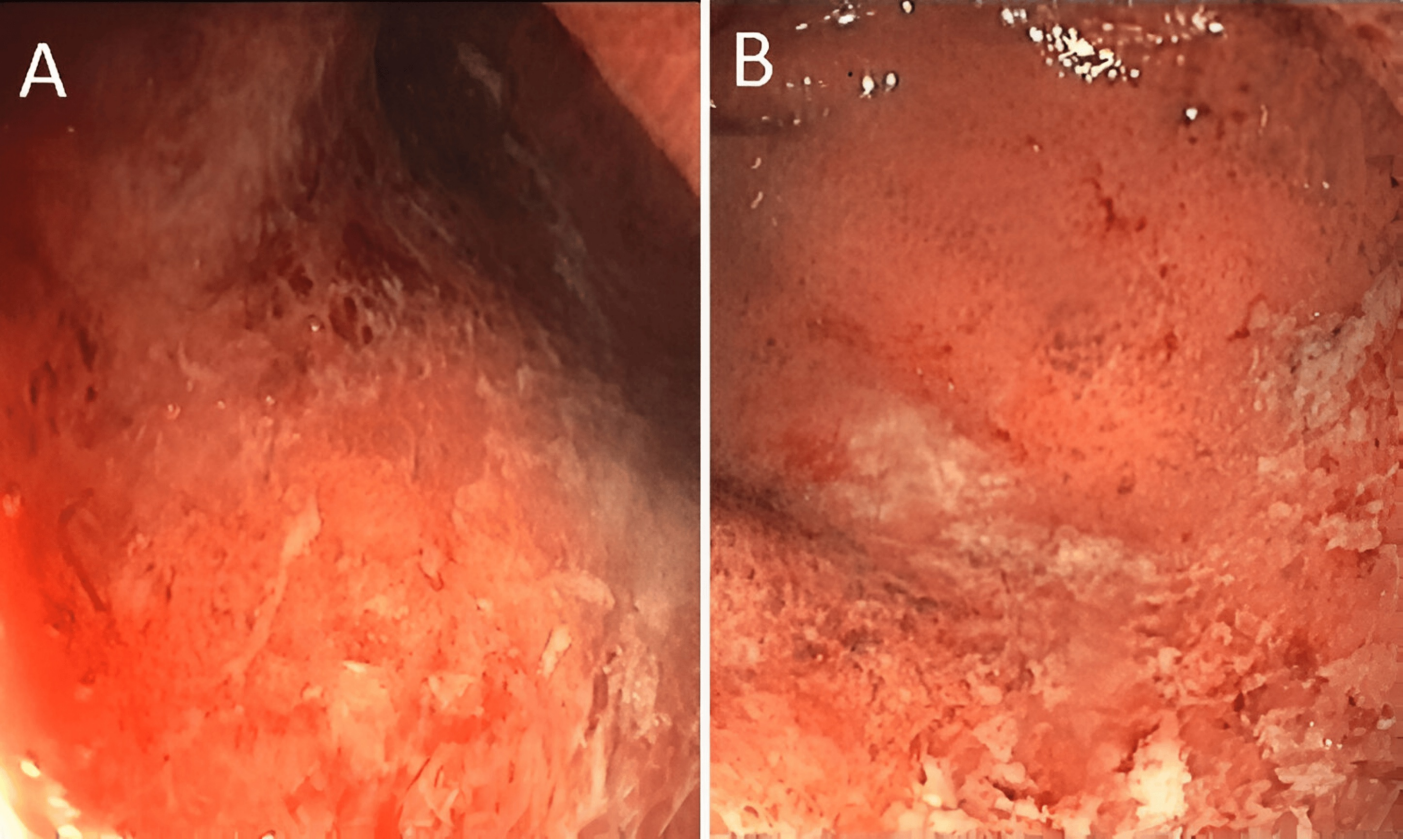
Colonoscopy showing diffuse mottling and friability of mucosa with no erosions or ulcerations

A subsequent colonoscopy performed, reaching up to 25 cm from the anal verge, exhibited mucosal changes characterized by diffuse mottling and friability, without any erosions or ulcerations (Figures 1A, 1B). Biopsies were procured for histological evaluation, which unveiled a lymphoplasmacytic inflammatory infiltrate, featuring small germinal centers and polymorphonuclear neutrophils penetrating the glandular crypts, alongside the formation of crypt abscesses. These inflammatory changes were localized to the mucosa and aligned with a diagnosis of UC.

**Figure 2:**
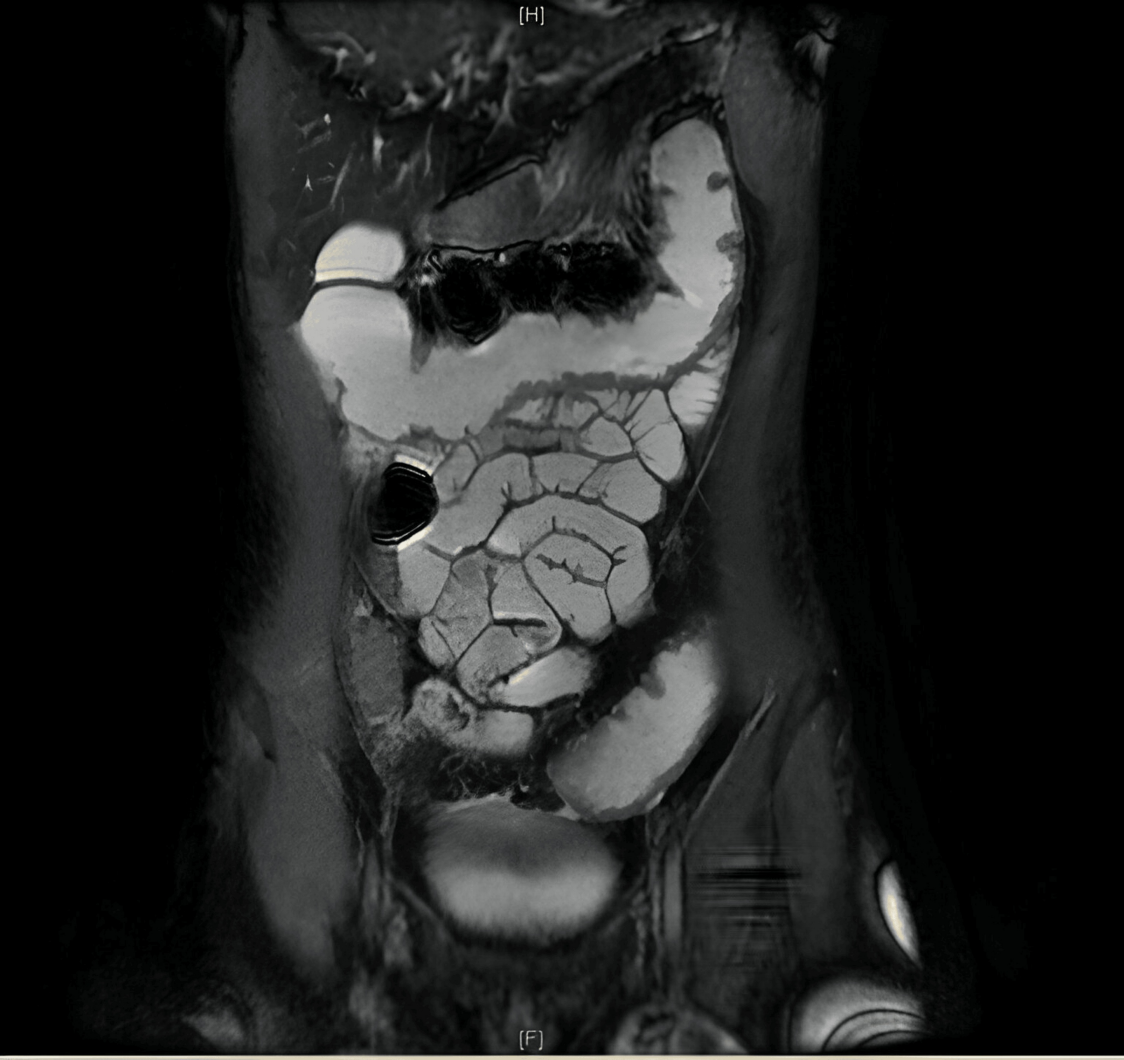
Thickening of the colonic parietal wall with no changes in the small intestine

Stool studies showed no presence of pathogens. Enterography magnetic resonance revealed mild thickening of the parietal wall diffusely present in the ascending, descending, and sigmoid colons without thickening of jejunal or ileal loops (Figure 2). The ingested contrast material displayed normal progression and distensibility of the small bowel loops.

Treatment was initiated with intravenous corticosteroids (hydrocortisone intravenous 100 mg 6-6 hours). During her hospital stay, the patient's condition significantly improved, culminating in her discharge on the fourth day of admission. She was prescribed a tapering regimen of oral prednisolone, along with mesalazine and iron supplementation.

**Figure 3:**
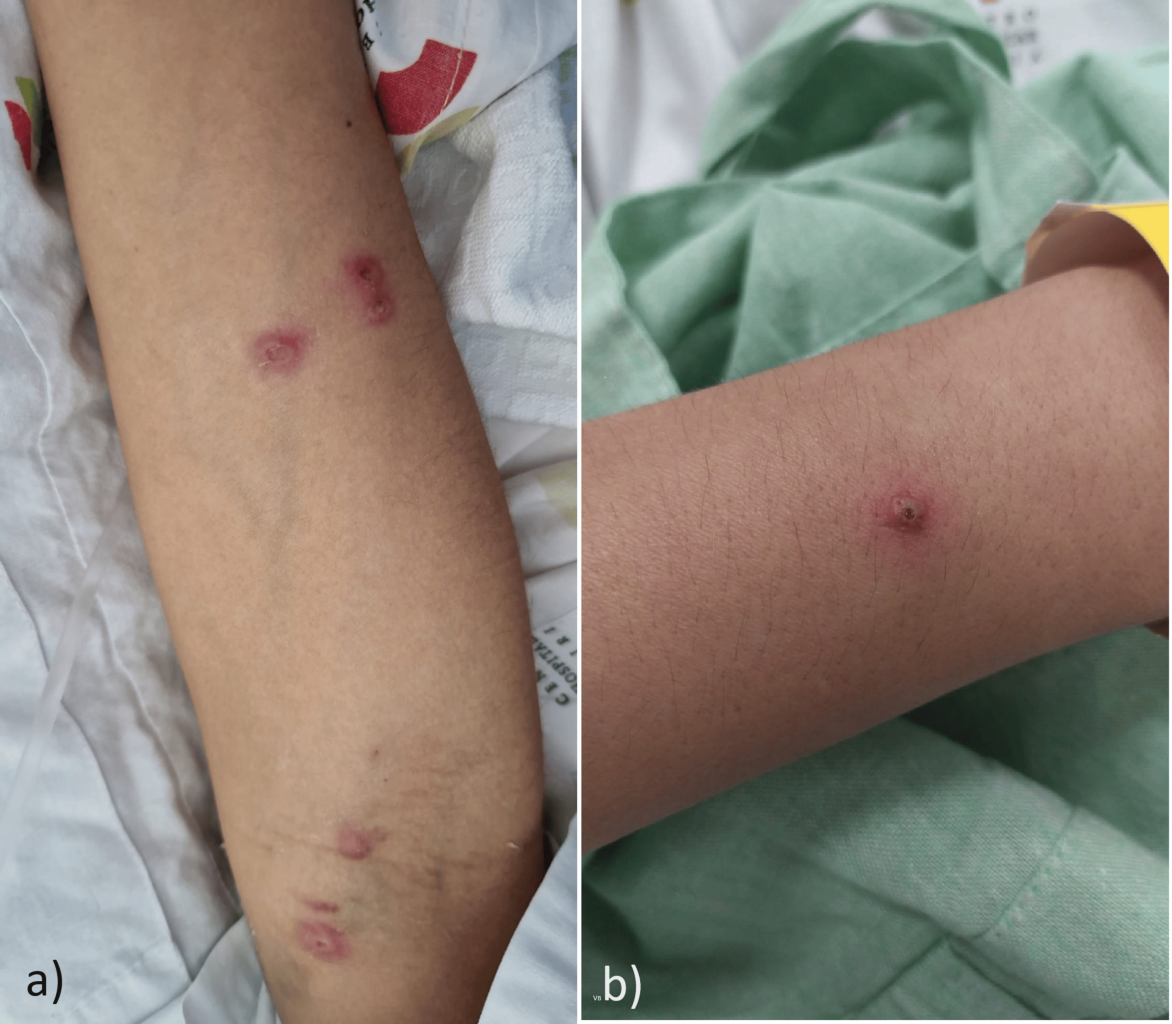
Firm, violaceous, 2-5 mm papulopustular lesions on the extensor surfaces of the upper right (a) and left (b) limbs

Following her initial discharge, the patient decided to discontinue the prescribed medication regimen. Recurrent episodes of symptoms ensued, prompting her to return to the hospital. She experienced a resurgence of fever, localized polyarthralgia affecting the knees and ankles, and a distinctive papulopustular rash on her arms, trunk, and lower limbs (Figure 3). Notably, lower back pain, dactylitis, enthesitis, photosensitivity, rash, and red eye complaints were absent. From a gastrointestinal perspective, the patient exhibited partial symptom improvement, characterized by a reduction to three daily bowel movements, diminished blood presence, and the emergence of more formed stools. During the physical examination, cutaneous assessment revealed a globally symmetric dermatosis, featuring firm, violaceous papulopustular lesions, measuring approximately 2-5 mm in diameter. Predominantly, these lesions were observed on the extensor surfaces of the upper limbs, with a few lesions dispersed on the trunk and lower limbs. Skin biopsies were obtained for histological examination, direct immunofluorescence, and tissue culture.

Laboratory investigations disclosed an increased white cell count (11.8 × 10^9^/L) with mild neutrophilia (8.3 × 10^9^/L) and a C-reactive protein level of 71 mg/dL. Results also indicated a hemoglobin of 13.1 g/dL and an erythrocyte sedimentation rate of 69 mm/h.

The patient received empirical ceftriaxone associated with oral prednisolone at a dosage of 20 mg, leading to partial resolution of the dermatologic lesions. Further autoimmune workup, including rheumatoid factor, antinuclear antibody, double-stranded DNA antibody, HLA-B51, complement components (C3 and C4), cytoplasmic and perinuclear antineutrophil cytoplasmic antibodies, HIV, and hepatitis B and C, returned within normal limits. Additionally, tests for cryoglobulins, anti-neutrophil cytoplasmic antibodies, hepatitis panel, anti-double-stranded deoxyribonucleic acid, anti-Ro/La/RNP, anti-Smith, anti-phospholipid antibodies, and anti-cyclic citrullinated peptide were negative.

On the fourth day of hospitalization, the patient experienced an exacerbation of diarrhea, and subsequent colonoscopy revealed mucosal ulcers, some with re-epithelialization, with adjacent pseudopolyps located in the transverse, descending, and sigmoid colon. One of these ulcers exhibited active pulsatile bleeding, which was managed with adrenaline and an endoclip. Prompted by these findings, intravenous corticosteroid therapy was initiated, leading to a significant improvement in the patient's condition.

**Figure 4:**
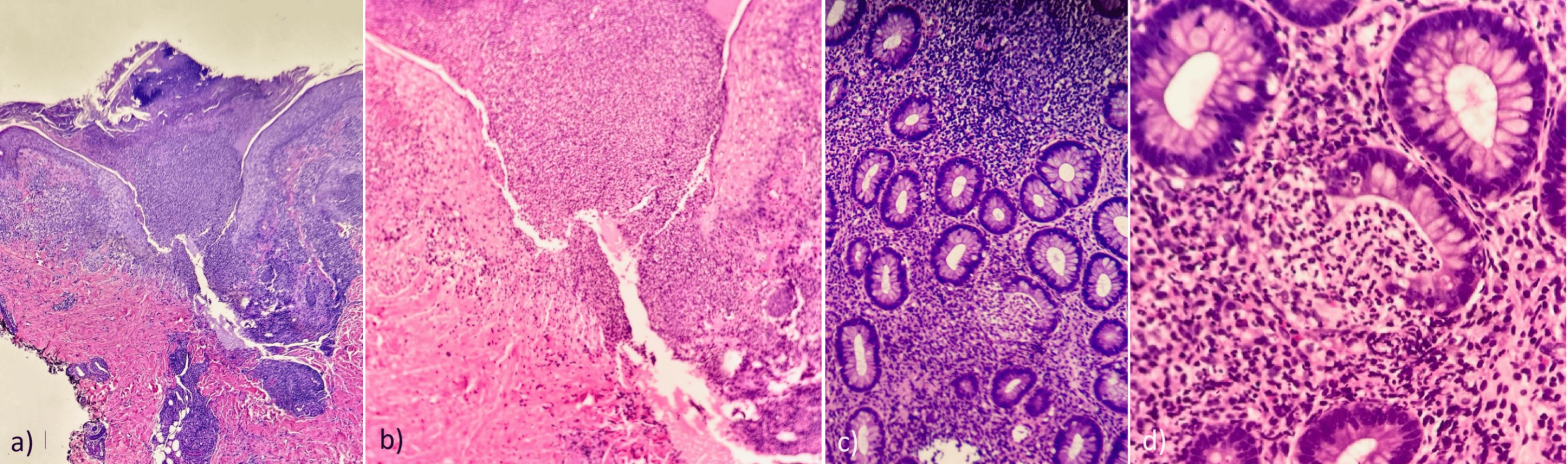
Histological examination of skin lesions showing superficial dermal edema (a, b) and a predominantly neutrophilic, perifollicular, and perivascular inflammatory infiltrate (c, d)

Bacterial culture from pustules yielded negative results, with both tissue culture and direct immunofluorescence also showing negative findings. Histological examination revealed superficial dermal edema and a predominantly neutrophilic, perifollicular, and perivascular inflammatory infiltrate (Figure 4). The findings are consistent with neutrophilic dermatosis, which, along with the history of UC disease and the current clinical presentation, led us to the diagnosis of BADAS in the setting of possible IBD.

The patient's course of treatment led to stabilization of the digestive flare-up and improvement in skin lesions one week after the initiation of corticosteroid therapy in addition to the referred antibiotic therapy. She was then discharged to outpatient care medicated with oral prednisolone (tapering scheme) and mesalazine.

During a two-week follow-up, the patient's lesions exhibited significant improvement, and at a three-month follow-up, complete resolution was noted, alongside improvement in gastrointestinal symptoms.

Due to the underlying disease process, the patient was proposed to be initiated on infliximab treatment, with efforts to gradually taper and withdraw prednisolone.

## Discussion

BADAS is a rare condition characterized by recurring cutaneous and articular manifestations in individuals with underlying gastrointestinal diseases. Initially coined as "bowel bypass syndrome" by Shagrin et al. in 1971, it was originally associated with bowel bypass surgery for obesity [2]. Recent insights have expanded its association to a spectrum of medical gastrointestinal disorders, notably IBDs like Crohn's disease and UC. These conditions serve as the principal contributors to BADAS, with bariatric surgery accounting for 63.7% of cases and IBD (most commonly UC) responsible for 24.7% of cases. Other conditions, including appendicitis, diverticulitis, and short bowel syndrome, have been sporadically associated with this syndrome [3-7].

Clinical manifestations

BADAS typically manifests as recurring, sterile vesiculopustular skin lesions accompanied by joint discomfort. The upper limbs are the primary site of involvement, accounting for 73.5% of cases, while occurrences in the lower limbs and trunk are less common. Facial symptoms are infrequent, noted in only 18.1% of cases, and isolated lip lesions have been sporadically documented [3]. These primary skin lesions manifest as erythematous macules, measuring 3-10 mm in diameter with indistinct borders. Over a subsequent 1-2-day period, these macules undergo induration and transform into papulopustular lesions, measuring 2-4 mm in diameter [4,5]. Joint manifestations in BADAS affect predominantly larger peripheral joints asymmetrically, including the knee, ankle, and elbow; however, symmetrical manifestations and involvement of the metacarpophalangeal and metatarsophalangeal joints have also been reported [8,9]. Gastrointestinal symptoms are more prominent in IBD-associated cases (67.9%) compared to non-IBD cases (23.5%) and appear to parallel IBD activity, similar to other neutrophilic dermatoses like Sweet syndrome but distinct from pyoderma [10].

In about 25% of cases, the cutaneous-articular symptoms of BADAS manifest over five years following the initial appearance of gastrointestinal disease or surgery. Nonetheless, the absence of gastrointestinal disease during the initial evaluation should not preclude consideration of a BADAS diagnosis, as it can be the primary presentation of IBD in 33% of IBD-related cases [3].

Diagnostic challenges

The diagnostic journey of BADAS remains complex due to the absence of established diagnostic criteria and its propensity to mimic several other dermatoses, including aseptic abscess syndrome, Sweet syndrome, pyoderma gangrenosum, panniculitis, and hidradenitis suppurativa [3,6]. The presence of fever and pustular lesions necessitates the exclusion of infectious causes such as gonococcal septicemia, systemic candidiasis, or subacute endocarditis. Negative cutaneous and blood cultures and direct immunofluorescence tests are consistent with BADAS [11].

Due to its variable clinical presentation, histological examination of skin lesions is essential to the diagnosis of BADAS. Histologically, a representative skin biopsy in BADAS typically reveals papillary dermal edema, concomitant with a robust, often perivascular neutrophilic infiltrate primarily localized to the upper dermis. Furthermore, evidence of leukocytoclasia may be discerned, while the characteristic features of fibrinoid vascular degeneration and leukocytoclastic vasculitis are notably absent [12,13].

Treatment approaches

While certain BADAS episodes may spontaneously resolve over several days, more severe or persistent cases require treatment [3,12]. Given the rare nature of BADAS, there are no established guidelines for its management, with current therapeutic approaches including systemic corticosteroids and antibiotics, either in isolation or combination. Antibiotics play a role in reducing gastrointestinal bacterial load and bacterial translocation, while corticosteroids modulate the immune response locally and systemically. Commonly utilized antibiotics include doxycycline and metronidazole.

However, remission after antibiotic and/or corticosteroid treatment tends to be transient [3]. To secure lasting remission, addressing the underlying disease is essential. In fact, remission can last up to three years with steroid and immunosuppressive therapy in IBD-related BADAS [3,13]. Management of underlying IBD involves an array of drugs, encompassing sulfasalazine, immunosuppressants (azathioprine, mycophenolate mofetil, cyclosporin), biological agents (infliximab, ustekinumab), and small molecules. Notably, some of these drugs, particularly anti-TNF-α agents like infliximab and, more recently, the IL-12/23 inhibitor ustekinumab, have become increasingly significant in these patients [1,13].

This case report highlights the diagnostic complexity and clinical heterogeneity of BADAS, presenting a unique diagnostic challenge. In this case, the patient's presentation with BADAS followed an initial diagnosis of UC. Regarding the diagnostic aspect, BADAS presents a challenging puzzle due to its resemblance to other neutrophilic dermatoses, which can result in misdiagnosis or delayed recognition. The interplay between BADAS and IBD activity further adds complexity to the clinical scenario, underscoring the necessity for comprehensive long-term management.

## Conclusions

This case report highlights the diagnostic complexity and clinical heterogeneity of BADAS in patients with IBD. With its varied clinical manifestations, potential mimicry of other dermatoses, and association with disease activity, BADAS presents a unique diagnostic and therapeutic challenge. Further case reports, case series, and retrospective studies are necessary to establish a consensus on diagnostic criteria and treatment guidelines.
